# Implementing telemonitoring in primary care: learning from a large qualitative dataset gathered during a series of studies

**DOI:** 10.1186/s12875-018-0814-6

**Published:** 2018-07-18

**Authors:** Janet Hanley, Hilary Pinnock, Mary Paterson, Brian McKinstry

**Affiliations:** 1 School of Health and Social Care, Edinburgh Napier University, Sighthill Campus, Sighthill Court, Edinburgh, EH11 4BN UK; 2 Allergy and Respiratory Research Group, Usher Institute of Population Health Sciences and Informatics, University of Edinburgh, Teviot Place, Edinburgh, EH8 9AG UK; 3 E-Health Research Group, Usher Institute of Population Health Sciences and Informatics, University of Edinburgh, Nine Edinburgh BioQuarter, Edinburgh, EH16 4UX UK

**Keywords:** Qualitative research, E-health, Primary care, Telemonitoring, Diabetes, Chronic Obstructive Pulmonary Disease (COPD), Hypertension

## Abstract

**Background:**

Telemonitoring for long term conditions such as hypertension and diabetes has not been widely adopted despite evidence of efficacy in trials and policy support. The Telescot programme comprised a series of seven trials and observational studies of telemonitoring for long term conditions in primary care, all with an explanatory qualitative component which had been analysed and published separately. There were changes to the models of care within and between studies and combining datasets would provide a longitudinal view of the evolution of primary care based telemonitoring services that was not available in the individual studies, as well as allowing comparison across the different conditions monitored. We aimed to explore what drove changes to the way telemonitoring was implemented, compare experience of telemonitoring across the range of long term conditions, and identify what issues, in the experience of the participants, need to be considered in implementing new telemonitoring systems.

**Method:**

Synthesis and thematic reanalysis of transcribed qualitative interview and focus group data from the Telescot programme adopting an interpretive description approach. All transcribed and coded text was re-read and data relating to the experience of the telemonitoring services, perceptions of future use and strategies for implementation were recoded into one consistent system. This was analysed thematically.

**Results:**

The combined dataset contained transcribed qualitative interview and focus group data from 181 patients and 109 professionals. Four major themes were identified, using data, empowering patients, adjusting the model of care and system design.

**Conclusion:**

Telemonitoring was valued by patients who found it empowering and convenient. This, combined with initial professional concern that increased surveillance may create dependency led to the development of a more patient led service. However, despite a number of initial concerns being addressed as the service evolved, primary care professionals identified a number of barriers to widespread routine adoption of telemonitoring, many of which could be addressed by improved system design.

## Background

Home telemonitoring in long term conditions has been defined as “*an automated process for the transmission of data on a patient’s health status from home to the respective health care setting.*” [1]. The potential for telemonitoring to improve the management of long term conditions such as hypertension, diabetes, heart failure and chronic obstructive pulmonary disease (COPD) by enabling more frequent measurement of key parameters without the need to visit healthcare premises seems logical. However so far, despite strategy documents advocating its use [2, 3] and numerous pilot studies, this form of telemonitoring has not been widely adopted [4]. Randomised controlled trials (RCTs) have had mixed outcomes [5], although the positive effect of blood pressure (BP) telemonitoring is now firmly established [5–7]. In contrast to the low uptake of telemonitoring by healthcare services, there is public demand for increased monitoring in long term conditions with a profusion of apps available for recording home measured health data. The experience of those taking part in telemonitoring trials (both patients and professionals) could provide valuable guidance in developing effective telemonitoring services which would be widely adopted. This paper reports an analysis of combined qualitative data from the Telescot series of seven primary care based telemonitoring studies [8–14] carried out between 2009 and 2014 mainly in Scotland.

Table 1 summarises the trials and observational studies in hypertension, COPD, heart failure and type 2 diabetes in the programme, all of which were analysed and published as separate entities [8–17]. All studies had a qualitative component [8–14]. Because it was a series of studies rather than one large implementation of telemonitoring, the models of care were flexible and their evolution was observed both within and between studies. The combined dataset therefore provides a longitudinal view of the evolution of primary care based telemonitoring services that was not available in the individual studies, as well as allowing comparison across the different conditions monitored. The consistent aspect of the telemonitoring services were that home monitored data were shared with the healthcare practitioner. The main evolving aspect of the services was increasing patient responsibility for acting on the home monitored data and reducing professional responsibility from constant surveillance of the data to periodic review unless contacted by the patient.

**Table 1:** The Telescot studies

	Telemonitoring Study	Design	Number in RCT	Primary Outcome	Patients interviewed	Healthcare providers interviewed
1.	COPD telemonitoring pilot [8]	Observational and qualitative study (27 participants). Monitoring symptoms, O2 saturation and FEV1)			20	25
2.	BP Telemonitoring trial (HITS) [9, 15]	RCT of BP telemonitoring with nested qualitative study	401	Systolic BP significantly lower in intervention group	25	20
3.	BP after stroke pilot trial [10]	Pilot trial of BP telemonitoring plus qualitative study	55		16 (plus 23 in 3 focus groups)	4
4.	COPD Trial [12, 16]	RCT of COPD telemonitoring with qualitative evaluation	256	No difference in days to first hospital admission	38	31
5.	Heart Failure [11]	Qualitative study			18	5
6.	Diabetes trial [13, 17]	RCT of blood glucose and BP telemonitoring with qualitative evaluation	321	HbA1c and systolic BP significantly lower in intervention group	23	10
7.	COPD “light touch” [14]	Observational and qualitative study (51 participants)			20	14 (8 in focus group)

The evolution of services is important. A number of intermediate level theoretical frameworks have been used to understand the dynamics and process of introducing new technologies into healthcare with the most frequently used being normalisation process theory with its important concept of ‘fit’ of the new technologies [18] and the diffusion of innovations [19]. Both these models highlight that ongoing use is unlikely if there is insufficient flexibility to enable new systems and care models to be adapted to meet the needs and expectations of the both patient and healthcare professional users (which in themselves will evolve as they become familiar with the technologies).

The experience of participants in trials and pilot studies can identify and illuminate the complexities and unexpected consequences surrounding home telemonitoring and guide decision making about future implementation. We therefore synthesised and re-analysed the qualitative data from all these studies with the aim of exploring what drove the changes in the way telemonitoring was implemented, comparing experience of telemonitoring across the range of long term conditions, and identifying what issues, in the experience of the participants, need to be considered in implementing new telemonitoring systems.

## Methods

### Context

Details of seven qualitative studies within the Telescot programme are shown in Table 1.

All the studies had been approved via the UK national research ethics service (references 12/EM/0359, 11/S1102/23, 10/S1102/160, 08/S1101/34, 08/S1101/38, 08/S1101/60) and participants gave specific written consent to the qualitative component [8–14]. This combined and comparative reanalysis of the qualitative data was carried out by the core research team for all these studies with no external transfer of data and was within the scope of the initial ethical approvals. The qualitative studies were all embedded in trials or observational studies gathering quantitative usage and outcome data except study 5 (heart failure) which was purely qualitative. In studies 1–6 telemonitoring was based on a surveillance model where the patient took their readings at agreed intervals and these were regularly reviewed by professionals (daily for COPD and heart failure, weekly- fortnightly for hypertension and type 2 diabetes). The professionals interpreted the data and contacted the patient if there were any concerns. In study1 (COPD pilot study) a call centre acted as an intermediary between the patient and their primary care practice and reviewing readings daily and contacting both the patient and practice according to a protocol if they breached any limits. (In this study healthcare professionals also initially had an associated advisory treatment protocol linked to the patient monitored parameters but this was dropped within a few weeks due to concerns that it would lead to overtreatment). In all other studies, the patients’ own doctor, nurse or therapist reviewed patient readings (only on request in study 7). The telemonitoring systems varied. The system used in the first two COPD (studies 1 and 4) and the heart failure studies (Study 5) as well as transmitting readings provided the patient with PC based educational material but no on-going record of their readings. In the hypertension and type 2 diabetes studies (studies 2,3 &6) readings were transmitted via Bluetooth/ mobile phone and patients had access to a web portal where they could view a record of their readings. The system also provided simple automated feedback via SMS text or email, but did not provide any educational material. In the final COPD study (Study 7) a much more patient-led approach was adopted and the professionals did not review the patients’ data unless the patient contacted them via a hotline and asked for advice. Due to technical issues patients initially kept a paper record of their readings which some opted to continue when the SMS based system became available. The professional interface for all systems required logging on to a separate system where readings submitted by all their patients could be reviewed.

### Qualitative approach

An interpretive description approach was adopted for qualitative studies throughout the programme [20]. This adopts a constructivist approach to knowledge using rigorous methods employed in more traditional approaches to qualitative research, but acknowledges that the purpose of the researcher is to gain insight which will be of use in a clinical context.

### Data collection

Qualitative data were gathered by JU, PF and SL (one male and one female social science researchers, one female nurse researcher, all educated to masters or doctoral level and previously unknown to the interviewee), through audio recorded semi structured face to face or telephone interviews and focus groups with patients, healthcare professionals and other stakeholders involved in the studies between 2009 and 2014. These were transcribed verbatim. Patients had been sampled purposively from participants in the associated trial or observational study to gain a maximum variation sample based on age, sex and location and all signed an additional consent for the qualitative study. In all cases the purpose of the interviews was stated as being to elicit the individuals’ experiences and opinions about telemonitoring and interviewees were aware that the interviewer was not involved in either providing the service or collecting quantitative outcome data. Full details about recruitment can be found in relevant papers [8–14]. Each qualitative dataset had been inductively coded by the relevant researcher using constant comparison to ensure consistency, with review by the full research team, and where possible by participants, to ensure viewpoints have been accurately represented. However, there had been no attempt to match coding frames across the different studies.

These data were anonymised and combined to form the dataset for this analysis. Four interviews with nurses involved in study 3 that had not previously been analysed were added to the dataset.

### Data synthesis and analysis

All transcribed and coded text was re-read and data relating to the experience of the telemonitoring services, perceptions of future use and strategies for implementation were recoded into one consistent system. Data relating to trial management issues or specific obsolete hardware were excluded. However data relating to software and hardware design which may be relevant to future systems were included. The initial re-reading and development of provisional codes was done on paper. Subsequently the recoded data were extracted from electronic (word) versions of the documents into tables. Steps to ensure this was a rigorous process included constant comparison and search for negative cases. The recoding was done by JH, and the recoding was reviewed and discussed by BM, HP and MP. Base codes were then grouped into sub themes and themes, again reviewed by the whole research team.

Data saturation, defined as no new themes or ideas arising in consecutive interviews when the research team were satisfied that a wide range of individuals had been interviewed, had been used to guide sampling in each of the studies (subject to minimum numbers agreed with funders). In this reanalysis of data the researcher re-read all the transcribed data but stopped adding data extracts to the coding frame when numbers became very large, having ensured that a wide range of individuals and all studies were represented.

Having identified the main themes and sub-themes emerging from this large recoded dataset, we mapped the data to Rogers [19] description of the characteristics of an innovation which are likely to lead to its adoption. The purpose of this additional step in the analysis was to illuminate both the aspects of telemonitoring which are likely to drive its adoption (including those which evolved through the series of studies) and those which require attention if they are not to become barriers to future adoption of telemonitoring.

## Results

The characteristics of the participants are summarised in Table 2.

**Table 2:** Characteristics of participants in the qualitative studies

	Patients		Professionals (number)			
Study	Mean age of patients	Proportion of male patients	Doctors (all general practitioners (GPs) except where stated)	Nurses	Therapists	Managers and Administrators
COPD pilot [8]	68	59%	4	6	3	12
BP trial [9]	61	60%	9	11		
BP after stroke pilot trial [10]	66	75%		4		
COPD Trial [11]	68	47%	3 GPs, 1 respiratory specialist	9	6	12
Heart Failure [12]	75	61%	1	4		
Diabetes trial [13]	60	65%	4	6		
COPD “light touch” [14]	67	50%		3	5	6

The thematic analysis led to four main themes: Data use, empowering patients, adjusting the model of care and system design. These themes and their subthemes are shown in Table 3 and presented below.

**Table 3:** Themes and sub themes

Theme	Associated sub themes
Using data	BP data • Measurement more trusted than surgery measurement • Patients and professionals confident to use as a basis for treatment change Blood sugar data • Not used by all professionals, some waited for HbA1c • Useful when treatment change contemplated • Sharing data motivated patients – diet and medication COPD data (O_2_ saturation, symptoms, FEV_1_) • Not discriminatory for severe exacerbation/ lots of false alarms • Complex, need to know the patient – can change fast • Overtreatment • Other symptoms Patients use of data • Previously not told their readings. • Understanding their normal • Reduced dependency on readings • Data providing motivation • Sharing data motivated patients • Not willing to keep manual record of self monitoring
Empowering patients	• Empowering patients ○ Use shared readings to actively negotiate care ○ Self management and reassurance • Confidence - patients/ families ○ Reassurance ○ Guardian angel/ access to care • Potential negative effects ○ Anxiety ○ Concern about dependency • Valuing convenience
Adjusting the model of care	• Scaling up • Workloads: ○ Fitting telemonitoring into practice workflows ○ Increasing or decreasing workloads • Communication channels with patient and within the team • Letting go • ‘How does this affect my role?’
System design	• Hardware • Reliability • Professional interface • Easy access via patient records • Easy two way communication • Feedback and decision support

### Theme 1. Data use

Telemonitoring provides patients and professionals with regular measurements of a range of symptom and physiological parameters. This generates a large volume of data and potential insights into the patient’s condition which had not previously been available. However, this series of studies demonstrated variation in the way these measurements were used. Regular home monitored blood pressure (BP) readings were useful to both patients and professionals who considered that they provided a more reliable assessment than BP measured occasionally in the surgery. Importantly they provided a trusted basis for treatment change which previously may have been delayed because of concerns about the representativeness of surgery based measurement.

*[prior to telemonitoring] …We’re all guilty of it...’we’ll just see how it goes, you know, maybe watch it. I’ll check it again tomorrow’ and they probably maybe sit on it a bit longer* (Practice Nurse 9, BP trial)

In contrast, blood glucose data were often not used in the management of type 2 diabetes by the professionals, who used HbA1c measured at surgery-based routine reviews as a basis for care, although there was general agreement that home monitored blood glucose data could be useful when medication changes were being considered or had been initiated. Blood glucose data was seen to be of greater use to the patients in guiding their day-to-day self-management of the condition. Regular data sharing had a motivating effect on patients in relation to diet and medication adherence, as they were aware that at some point the readings may be reviewed.

*But knowing that everything’s going to be looked at on a regular basis by somebody else, it’s actually, it’s not bad to have a policeman somewhere ….* (Patient 6, Diabetes trial)

The motivating effect was also seen to some extent with blood pressure readings.

*When I was taking the blood pressure I couldn’t bear looking at 140, 150 over 110 and I wanted to just be able to see better readings in a way. So over the summer as well, starting to get more walking exercise, that kind of thing…. I didn’t want beta blockers because they had various side effects …so it did spur me on to look for alternative…* (Patient 2, BP trial)

The purpose of telemonitoring in COPD was to spot signs of impending exacerbations and intervene early. In practice the data were difficult to interpret and staff reported many false positives. Daily monitoring by a professional was not thought to be useful in preventing hospital admission as healthcare staff were not convinced enough was known for clear signals in the data to be recognised.

*this patient’s peak flow’s up or down, all this... oxygen saturation might have dropped or increased a little bit, but we are not quite sure what that... means in the clinical context with all the other conditions.* (GP 11, COPD trial)

There was also concern amongst professionals that acting on the additional data provided by telemonitoring could lead to overtreatment. This was mainly seen in COPD although there was also an example relating to blood pressure management.

*You can’t just continually prescribe antibiotics for these people. …. But then if you’re scoring 9 - and some of these people can go down SO quickly*… (Practice Nurse 1, COPD pilot study)

*He was quite surprised when I said “We’re aiming for a target here of 130/80.” Because his blood pressure readings have been 148/86, which really for most people they would look at that at 92 years old and think that’s quite acceptable* (Practice nurse 5, BP following stroke)

Although healthcare staff did not find COPD data particularly useful, patients used their data, particularly oxygen saturation, to guide day-to-day management of their health. Although they found it difficult to tell if changes in their respiratory symptoms were leading to acute exacerbation, the steady oxygen saturation readings and absence of non respiratory symptoms they associated with the onset of an exacerbation (such as sore legs) gave them confidence to go about their normal activities.

*Sometimes I wouldnae (would not) go out for six or eight weeks. Now I know and I take my reading, I can go out any day I want. I have my confidence back and that is what it’s done for me* (Patient 10, light touch telemonitoring)

### 2. Empowering patients

Overall, generating and recording the telemonitoring data empowered patients. They now had information about their health which previously had been in the domain of the healthcare providers and frequently had not previously been shared with them. They felt more able to participate actively in consultations and manage their own health.

*It’s certainly given me more meaningful data to speak to the doctor rather than, “Well, I think my BP has probably gone up.”* (Patient 6, BP trial)

*He’s more aware of his symptoms. He understands when he should be seeking help, so he’s not just tending to phone up every time that he gets a cough or a bit more breathless because, unless he’s got all the symptoms, that’s when he would contact us. So it’s helped him to manage his own condition better* (Nurse 17, COPD trial)

Most patients found the ability to take their own readings reassuring, particularly when they were normal and, as noted above, for some it increased their confidence. Another source of confidence, particularly for those with COPD, was that the telemonitoring data enabled them to negotiate prompt access to care. The convenience of taking readings at home, particularly blood pressure readings, was also highly valued.

*For me personally just taking it in the house, rather than go up there and sit with the coughs and splutters* (patient 1, BP following stroke study)

Some patients felt empowered to take a more proactive approach to their health care based on their readings and made appointments rather than waiting for professionals to contact them, if necessary circumventing the supporting advice services. For example, if patients felt they needed a new prescription and thought the nurse or therapist who managed their telemonitoring data could not prescribe, they would go directly to a GP. On occasions, this disrupted the usual workflows and communication channels within the practice, and illustrated the need for telemonitoring data to be easily available with the medical records.

*… so I’m not even going to bother with her [member of respiratory team], … I’ll just use the wee oxygen monitor and just use it every day,… if it goes below two for two or three days, then I know to go along to the doctor.* (Patient 7, COPD light touch study)

However, although many patients described taking a proactive approach to their long term condition, occasional review of the data by a healthcare professional was appreciated, particularly when blood pressure or blood glucose readings were in the normal range but could be better.

*And then I was getting higher readings, and I thought… I thought, ‘Oh well somebody’s monitoring it. If there’s something dangerous, somebody’s going to tell me.’ I hope!* (Patient 28, diabetes trial)

All patients taking part in these studies were eligible because their condition was sub-optimally controlled, but as control improved they tended to reduce the frequency of their readings. This was particularly noticeable in the ‘light touch’ COPD telemonitoring where people tended to monitor until they recognised their usual pattern of measurements and then stopped until they felt something was wrong.

Although most patients were empowered and reassured by telemonitoring, a very small number were made anxious by it. Of all the patients interviewed, only one reported increased anxiety, but several professionals commented on this possibility. Across all the early studies, professionals were concerned that the surveillance model of care, in which professionals reviewed and interpreted the submitted data, would lead to anxious patients becoming dependent.

*…they like it because they’re getting somebody that’s looking at their symptoms every day of the week… And that’s why we need to be careful over this group of patients who are already quite anxious anyway, I think we’re making a dependency (*Therapist 8, COPD trial)

The model of care used in the final study (study 7) where the monitoring data provided by COPD patients was only viewed if the patients were concerned and contacted the helpline addressed the concerns about dependency.

### Theme 3. Adjusting the model of care

This theme was generated largely by the professionals and service providers interviewed. Many professionals were happy to contribute to research by participating in the trials where only a small proportion of their patients were using telemonitoring but would not have been willing to implement the systems widely until their intertwined concerns about both the care model underpinning telemonitoring and design of telemonitoring systems were addressed. Associated with these were concerns about workloads, workflows and role.

*I think at the moment it is totally appropriate for us as practitioners to do [tele-monitoring], but again I think it’s because the numbers are manageable. I wonder how it would be if we had 60 or 70 or 80 patients with telehealth … (*nurse 13, COPD trial)

The care model employed in the COPD pilot study which used a call centre to review telemonitoring data was changed in subsequent trials to one where the GP practice or specialist respiratory service was responsible for daily monitoring. Generally this was preferred by many practitioners who felt that it was important to know the patient, although not all thought this essential.

*I would probably think it’s better done within the practice … I know you have the team that they can contact, but rather than taking it outwith the practice, I think they’re probably more comfortable with the practice itself and the people that they know here*. (Practice nurse 9, diabetes trial)

However, as noted above, there was concern that the surveillance model of telemonitoring employed in studies 1–6 could create dependency and professionals taking part in the early trials expressed a preference for a model which shifted responsibility to the patient to a greater extent.

*… if your weight goes up, you get these symptoms, you must phone, you have to take responsibility for your health and in one way you’re taking that responsibility away from them cause all they’ve to do is to slap a machine on every day… and not necessarily think about it so much...*(Nurse 2, heart failure study)

*I think, patients are more and more, you know, will be self-managing,….* (Practice nurse 2, diabetes trial)

Professionals were also concerned about workloads. Both patients and professionals thought that in the longer term telemonitoring would reduce the number of visits required but during the trials professionals experienced increased workloads for a number of reasons (except in study 7 where use of the hotline was lower than expected). Professionals, mainly practice nurses, had to find regular time to log into systems to monitor the data across the patient group with the unprocessed stream of raw data taking some time to interpret. This did not fit with their normal workflows where reports relating to individual patients arrived mainly through a single electronic transfer system and could be added to the electronic medical record after review. Some professionals tried to fit logging on to the telemonitoring systems into their normal workload between patients; others put a specific time aside. However these studies took place during a well documented time of increasing pressure on primary care in the UK and finding time to log in and review telemonitoring data became more difficult.

*I think we weren’t as busy with [previous study] and that’s why it didn’t feel quite as intense but this time we just haven’t had… we have catch up slots and admin time and we’re squeezing people into that time, so all of time is just being eaten up by appointments at the minute*. (Practice nurse 5 BP following stroke trial)

However, they also found that with experience, and forced by the pressure of work, they were able to reduce the level of supervision and contact with patients without adverse effects.

*I was a lot more involved with the patients [in previous study], I seemed to communicate more with patients than I did this time.* (Practice nurse 1 BP following stroke trial)

Although there were concerns about the additional workload generated by telemonitoring there was also recognition that there were some elements of personal practice which professionals needed to let go for telemonitoring to make an efficient contribution to care. This included stopping the reliance on clinic measurements and using the patients’ self-measurements. For example, in all three studies involving BP monitoring incidents were reported where patients had high self-monitored BP, but treatment decisions had been made on a single measurement taken when they visited the surgery.

*…So they’re coming in to see the doctor, the doctor takes their BP, one forty five ninety; ‘oh, that’s fine, what are you worried about?’ ... And then you go and you look at it the next week and you think; they’ve seen the doctor and yet their BP’s still really high* (Practice Nurse 11 BP study)

On a practical level, this problem was potentially caused by the patients’ readings not being available from within the electronic medical record (see data on system design below).

A small number of professionals were concerned that although telemonitoring could potentially reduce primary care workloads it may change individuals’ roles in a way they would not like.

*It is not my type of medicine I have to say. I don’t monitor patients remotely by means of electronic equipment. I would rather actually see a patient, discuss them on telephone, to make a clinical decision about seeing them, assessing, examining them, knowing their history.* (GP11, COPD trial)

Reducing the level of face to face contact with the patients was a concern for professionals, but this concern was not universally shared by patients, some of whom experienced the non face to face contact as additional and efficient input.

### Theme 4. System design

Contacting patients by phone in relation to their telemonitoring data was found to be very time consuming and the need for agreement about who (professional or patient) should initiate contact if readings were abnormal was raised. Both patients and professionals commented that the telemonitoring systems lacked the ability to send ad hoc messages so there was no easy way of letting patients know that a professional had reviewed and was happy with their data leading to some patients wondering if anyone was looking at their records.

*I’ve heard from [healthcare professional’s name], a couple of times he has phoned... The first time in response to you having pressed the opposite answer to every morning’s answer just to see what would happen. I thought, you know, I’m doing this and I don’t know if anybody is looking at it…* (Patient 11, Heart Failure study)

By the final study (study 7) the issues relating to contact had largely been resolved by making patients responsible for initiating contact if they were concerned about their readings and the provision of a telephone hotline.

Another aspect of system design which caused concern was the inability to access the telemonitoring data via the electronic the medical records without a separate log in, which complicated consultations. Unreliable systems, inadequate maintenance of peripherals, poorly designed professional interfaces which were time consuming to navigate, and lack of intelligent decision support also caused frustration.

*there was a problem with the system. So again that was a time consuming thing that I had to chase up….* (Practice nurse 5, BP following stroke trial)

Although professionals thought that systems could be better designed to fit with their own workflows rather than them adjusting their workflows to the system, some also found that they needed to reconsider how information flowed within the practice to use the patient generated data efficiently and coherently.

### Mapping the data to the characteristics of an innovation likely to influence its adoption

The data relating to the experience of patients and professionals in trials of telemonitoring were mapped to Rogers [19] list of characteristics of an innovation likely to influence its adoption (see Table 4). This provided a useful framework for summarising the aspects of telemonitoring which would be likely to encourage its adoption, and those which, based on the experience in these trials, would need careful consideration in any future implementation.

**Table 4:** Mapping to the characteristics of an innovation likely to affect its adoption

Characteristic of the innovation (from Rogers (1995) The diffusion of innovations) [19]	Positive findings from this study	Negative findings from this study
Relative advantage (the perceived efficiencies gained by the innovation relative to current tools or procedures)	• Convenience • Empowerment • Confidence • Motivation • Better measurements • Could potentially reduce workloads	• Anxiety/dependence in small numbers of patients • Professional concerns about creating dependency • Concern about workloads
Complexity/ difficulty to learn	• Most patients found telemonitoring easy	• Professional interfaces complex • No clear signals for professionals in COPD data • Lack of intelligent decision support
Compatibility with the pre-existing system	• Preference for adoption within current system which influenced later implementations	• Lack of fit of professional system interfaces with workflows within practices • Concern about roles
Trialability or testability	• High –particularly for patients	
Potential for reinvention (using the tool for initially unintended purposes)	• Patients used data to manage day to day activity	
Observed effects	• Higher workloads – reduced with learning and external drivers such as pressure on the system. • Patients using data to support self management. • Increased responsibility for the patient in study 7 was acceptable	• Limitations and extra work created by system design • Empowered patients bypassing nurses or therapists, particularly with regard to prescribing

## Discussion

This analysis explored the experience of patients and professionals in trials of telemonitoring in the management of a range of long term conditions in primary care in the UK, mainly in Scotland. The additional value of the combined dataset is that provides a longitudinal focus on telemonitoring services which were evolving from trial to trial based on experience in the previous trial.

The experience of implementing telemonitoring for long term conditions challenged the simple model in which the patient takes measurements and shares them telemetrically with the professional who then adjusts treatment based on the readings. Not all data could be used in this way, particularly in COPD where no strong and reliable signal of the early stages of an exacerbation could be identified. However, generating their own measurements empowered patients who used them in managing their health and accessing appropriate healthcare, although they also valued occasional routine review of their data by primary healthcare professionals. Additionally professionals were wary of the initial surveillance model of care which they thought would increase dependency on health services and the evolution of the services was towards a more patient led approach.

Both patients and professionals found it more efficient for telemonitoring to be situated in existing primary care services. However, professionals found that system design limited their ability to integrate telemonitoring into normal primary care workflows and there were concerns from a small number of healthcare professionals that a reduction in face to face contact with the patient would negatively impact on their role. Mapping the experiences of both patients and professionals against the characteristics influencing the adoptability of a technology from the diffusion of innovations model [19] suggest that from the patients’ viewpoint adoption of telemonitoring is highly feasible. Challenges relating to the usability of systems by primary care professionals need to be considered if there is to be widespread adoption of telemonitoring in primary care in the UK.

### Strengths and limitations

The strengths of this study are that it is based on a large dataset and the themes emerged strongly. The analysis of the combined dataset showed that, apart from a few very condition-specific issues, there was substantial commonality in the experience of telemonitoring across the different conditions and different technologies used. Indeed the technology used to enable the telemonitoring service appeared to be of secondary importance to the redesign of the care model. Although qualitative data is situated in context and not generalisable, the dataset incorporates wide experience of using telemonitoring in long term conditions and the learning is likely to be transferable. However, the studies were limited to primary care in the UK (mainly Scotland) and may not reflect responses to telemonitoring in other health care systems. A further limitation is that the interviewees were participating in trials so their exposure to telemonitoring was relatively short term. The semi structured interview guides were designed separately for each study and although similar were not fully consistent across the studies. A strength is that despite this, the themes reported emerged strongly across the studies.

### Interpretation in the light of published literature

No comparable qualitative studies based on this level of telemonitoring experience across an evolving telehealth service have been found. The European Union *Renewing Health* project [21] which involved over 7000 patients across Europe gathered qualitative data from patients, and reported a similarly positive response from them. Evaluation of this project was guided by the model for assessment of telemedicine (MAST) framework [22] which does not include healthcare professionals’ responses, although one recommendation of the Renewing Health project was that the healthcare professionals viewpoint should be included in MAST. Our findings support this, suggesting that it is the professional experiences of systems which are currently likely to prevent them from being widely adopted. The UK based Whole Systems Demonstrator project included a qualitative study amongst healthcare professionals [23] which reported findings broadly consistent with those reported here, apart from their finding of a much more negative response from GPs. This may have been because in some areas the nurse-led telemonitoring service was separate from the GP practices. The wider literature suggests that grafting a new technology in pilot form to an existing management system seldom produces time savings in the short run [24] and in general needs it to be a good ‘fit’ with current systems before it is normalised [18]. Greenhalgh [25] warns that forcing professionals to drop their existing routines and adopt new, seemingly more efficient, ones may inadvertently create inefficiency, which was what was reported by the professionals in this study. However, subsequent research within the Telescot programme has suggested that by working with the healthcare professionals it would be possible to develop telemonitoring systems which could be integrated seamlessly within current primary care systems and working practices [26].

## Conclusion

This synthesis of the qualitative findings of a series of telehealth studies found that telemonitoring was generally valued by patients with a range of long term conditions who found it to be both empowering and convenient. This, combined with professional concern that the surveillance model of telemonitoring may create dependency led to the development of a more patient led service. In contrast, despite a number of initial concerns being addressed as the service evolved, primary care professionals identified a number of barriers to widespread routine adoption of telemonitoring. In particular professional facing aspects of the systems tested did not fit with General Practice workflows and all were perceived to be excessively time consuming. If the potential benefits of telemonitoring are to be achieved on a widespread basis, system designers and system evaluation must address the needs of both patients and practitioners.

## Abbreviations

BP: Blood pressure
COPD: Chronic obstructive pulmonary disease
RCT: Randomised controlled trial
